# Implicit Processing of Visual Emotions Is Affected by Sound-Induced Affective States and Individual Affective Traits

**DOI:** 10.1371/journal.pone.0103278

**Published:** 2014-07-29

**Authors:** Tiziana Quarto, Giuseppe Blasi, Karen Johanne Pallesen, Alessandro Bertolino, Elvira Brattico

**Affiliations:** 1 Cognitive Brain Research Unit, Institute of Behavioral Sciences, University of Helsinki, Helsinki, Finland & Finnish Centre for Interdisciplinary Music Research, University of Helsinki, Helsinki, Finland; 2 Psychiatric Neuroscience Group, Department of Basic Medical Sciences, Neuroscience and Sense Organs, University of Bari, Bari, Italy; 3 The Research Clinic for Functional Disorders and Psychosomatics, Aarhus University Hospital & Interacting Minds Centre, Aarhus University, Aarhus, Denmark; 4 pRED, Neuroscience DTA, Hoffman-La Roche, Ltd., Basel, Switzerland; 5 Brain & Mind Laboratory, Department of Biomedical Engineering and Computational Science, Aalto University School of Science, Helsinki, Finland; University of Toyama, Japan

## Abstract

The ability to recognize emotions contained in facial expressions are affected by both affective traits and states and varies widely between individuals. While affective traits are stable in time, affective states can be regulated more rapidly by environmental stimuli, such as music, that indirectly modulate the brain state. Here, we tested whether a relaxing or irritating sound environment affects implicit processing of facial expressions. Moreover, we investigated whether and how individual traits of anxiety and emotional control interact with this process. 32 healthy subjects performed an implicit emotion processing task (presented to subjects as a gender discrimination task) while the sound environment was defined either by a) a therapeutic music sequence (MusiCure), b) a noise sequence or c) silence. Individual changes in mood were sampled before and after the task by a computerized questionnaire. Additionally, emotional control and trait anxiety were assessed in a separate session by paper and pencil questionnaires. Results showed a better mood after the MusiCure condition compared with the other experimental conditions and faster responses to happy faces during MusiCure compared with angry faces during Noise. Moreover, individuals with higher trait anxiety were faster in performing the implicit emotion processing task during MusiCure compared with Silence. These findings suggest that sound-induced affective states are associated with differential responses to angry and happy emotional faces at an implicit stage of processing, and that a relaxing sound environment facilitates the implicit emotional processing in anxious individuals.

## Introduction

Perception and interpretation of facial expressions [1] are vital nonverbal sources of information [2]. The ability to recognize and label emotions contained in facial expressions is modulated by current affective states [3]. Bouhuys, Bloem, & Groothuis [4] induced temporary variations in subjects' mood with sad or happy music, which led to a modification of the labeling of emotionally ambiguous faces. In particular, participants labeled faces as happier when they were in an elated mood and as sadder when a negative mood was induced. Similarly, induced recollection of emotional autobiographical memories significantly affected the number of emotional faces detected by subjects, increasing the detection of frowning faces after sad mood induction and of happy faces after positive mood induction [5]. Together with other similar findings [6] [7] [5], these studies suggest that individuals in a negative affective state recognize more negative stimuli compared to positive or neutral stimuli, whereas individuals in a positive affective state tend to be more accurate in recognizing positive targets.

Stable individual emotional dispositions, i.e. affective traits, also play a fundamental role in the recognition of emotional stimuli. For example, individuals with higher trait anxiety tend to misclassify neutral expressions as angry and are more sensitive to threatening faces [8], [9]. The relationship between affective traits and affective states is not entirely understood. It has been argued that some affective traits may represent long-term *sequelae* of affective states [10], [11]. On the other hand, some affective states are more likely to be achieved by people with specific affective traits [12], [13]. For example, there is evidence that extroverts and neurotics may be differentially sensitive to stimuli that generate positive and negative affect, respectively [14]. Neurotics present heightened emotional reactivity to negative mood induction, whereas extroverts compared with introverts are more emotionally reactive to positive mood induction [12]. Similarly, individuals with higher trait anxiety seem more likely to adopt a threatening interpretation of ambiguous information, when surrounded by a negative environment [15]. These studies allow us to hypothesize that affective traits may be associated not just with different emotional recognition but also with different emotional reactivity to affective state induction techniques. It is possible that affective states and affective traits interact and integrate to produce complex behavioral patterns in a predictable way. Knowledge in this domain could potentially be used to facilitate adaptive behavior, e.g. by presenting suitable environmental cues to modulate the expression of dysfunctional affective states [16], [17].

Affective states or mood can be successfully regulated with drugs that operate directly on different neurotransmitters in the brain as well as via stimuli in the environment that impact our senses and, although in a less direct manner, induce plastic changes in the brains' circuits. In this perspective, music represents an affective state induction technique [18], which is non-intrusive and easily applied. Indeed, music is used by most people for self-regulation of emotions in everyday life [19], [20], and its power to reduce tension, modulate mood, and raise energy has been widely documented [19], [21], [22]. Music is used to modulate affective states in a large number of neurological and psychiatric disorders [23], and brain traumas [24]–[26]. Naturally, the effect of music on listeners is mediated by music culture and individual preferences, which are in turn correlated with age, gender, personality, listening biography, and cognitive style [27], [28].

The profound effects of music on transient affective states are documented in relation to explicit emotional processing of facial stimuli, which was biased towards the emotional valence of the musical stimuli. Specifically, activity in cortical brain regions involved in auditory and emotional processing increased during recognition of a positively valenced face when positively valenced music had been presented either simultaneously or as prime [29]–[31]. However, these studies focus only on the conscious labeling of facial expressions. Moreover, the previous studies utilize only music having sad or happy/pleasant connotations, not allowing for generalization to other sound environments that might be relevant for pathological conditions within the anxiety disorder spectrum (such as noise or relaxing natural sounds). Hence, very little is known about how relaxing or irritating sound environments might influence the implicit emotional processing of faces in healthy subjects, i.e. occurring when faces are presented to the subjects without any explicit emotional recognition or labeling task [1], [32]–[36]. Even less is known about whether the affective properties of relaxing or irritating sound environments impact all individuals similarly or whether some individuals with defined personality and affective traits are more affected than others by one kind of environment over another.

In the present study, we tested the hypothesis that relaxing vs. irritating sound environments and the derived affective states affect emotional responses during implicit processing of facial expressions. Specifically, we hypothesized that reaction times in a task involving implicit processing of facial emotions would be reduced or increased depending on the sound stimulation accompanying the task, indexing the engagement of neural resources to the implicit emotional processing of the faces stimuli [34], [37].

Moreover, we hypothesized that trait anxiety and emotional control would interact with the effects of the sound background. Particularly, we expected that subjects with greater emotional control and anxiety would be differentially sensitive to the effects of relaxing vs. irritating sound stimulations, reflecting in this way different sensitivity to the affective state induction technique used in this study.

## Methods

### Ethics statement

The present study was approved by the Comitato Etico Indipendente Locale of the Azienda Ospedaliera “Ospedale Policlinico Consorziale” of Bari. Informed written consent was obtained from all participants before participation after the procedures had been fully explained to them.

### Subjects

Thirty-two healthy subjects (11 males; mean age: 26.8±3.7) participated in the study. Inclusion criteria were absence of psychiatric disorders, absence of pharmacological treatment or drug abuse in the last year, and absence of any significant medical condition including hearing deficits. Subjects with musical training lasting more than 10 years were excluded from the study, because they were considered as musicians and musical expertise might represent a confounding factor in the analysis. This threshold was based on previous published articles investigating behavioral and neural differences between musicians and non-musicians [38]–[40]. Handedness (Edinburgh Inventory: 0.76±0.24) and socio-economic status (Hollingshead Four Factor Index: 31.87±13.31) were also measured (see Table 1). Independent-sample t-tests and χ^2^ test were used to compare demographics between groups divided for the affective traits investigated in the study. Repeated-measures ANOVAs were used to compare accuracy at the gender recognition task between the affective traits' groups and across all the experimental conditions.

**Table 1:** Demographic data.

	All	High STAI X2	Low STAI X2	High EC	Low EC (n = 16)
	(n = 32)	(n = 16)	(n = 16)	(n = 16)	
**Age, years**	26.8 (3.7)	26.5 (3.4)	27.1 (4)	27.5 (4)	26.1 (3.3)
**Gender, ** ***n***					
** Male**	11	5	6	7	4
** Female**	21	11	10	9	12
**Handedness**	0.8 (0.2)	0.8 (0.2)	0.8 (0.2)	0.8 (0.2)	0.8 (0.2)
**Hollingshead index**	31.9 (13.2)	33.9 (13.9)	29.9 (12.2)	31.3 (11.7)	32.5 (14.6)

The table shows the demographic data of participants both as a whole group or divided by high/low STAI-X2 and EC groups. The groups did not significantly differ in any of the demographic variable investigated (all p>0.1).

### Sound stimuli and extraction of acoustic features

The affective state of participants was positively modulated with a 5-minute representative cross-section of a contemporary musical piece entitled “MusiCure” composed by Niels Eje [41], designed for therapeutic and relaxation purposes. MusiCure includes not only classical instruments playing a clear melodic line and harmonic accompaniment but also natural and environmental sounds, such as animal vocalizations, the sounds of weather and other natural elements (Sound S1). Since 1999, MusiCure has been tested in surgery rooms, post anesthesia care units, neonatal, and psychiatric wards with documented effects on patients' wellbeing [42], [43]. Conversely, to induce a negative affective state, we generated a Noise stimulus sequence, that maintained the main acoustic characteristics and structure of MusiCure. Using the MIRToolbox (freely available toolbox of Matlab implemented at the University of Jyväskylä, Finland [44]) we extracted the amplitude spectrum of MusiCure and applied it to a white noise signal that was amplified in the average frequency range of MusiCure. The resulting Noise stimulus was balanced to MusiCure in the amplitude content and also partially in the pitch content (Sound S2).

**Figure 1:**
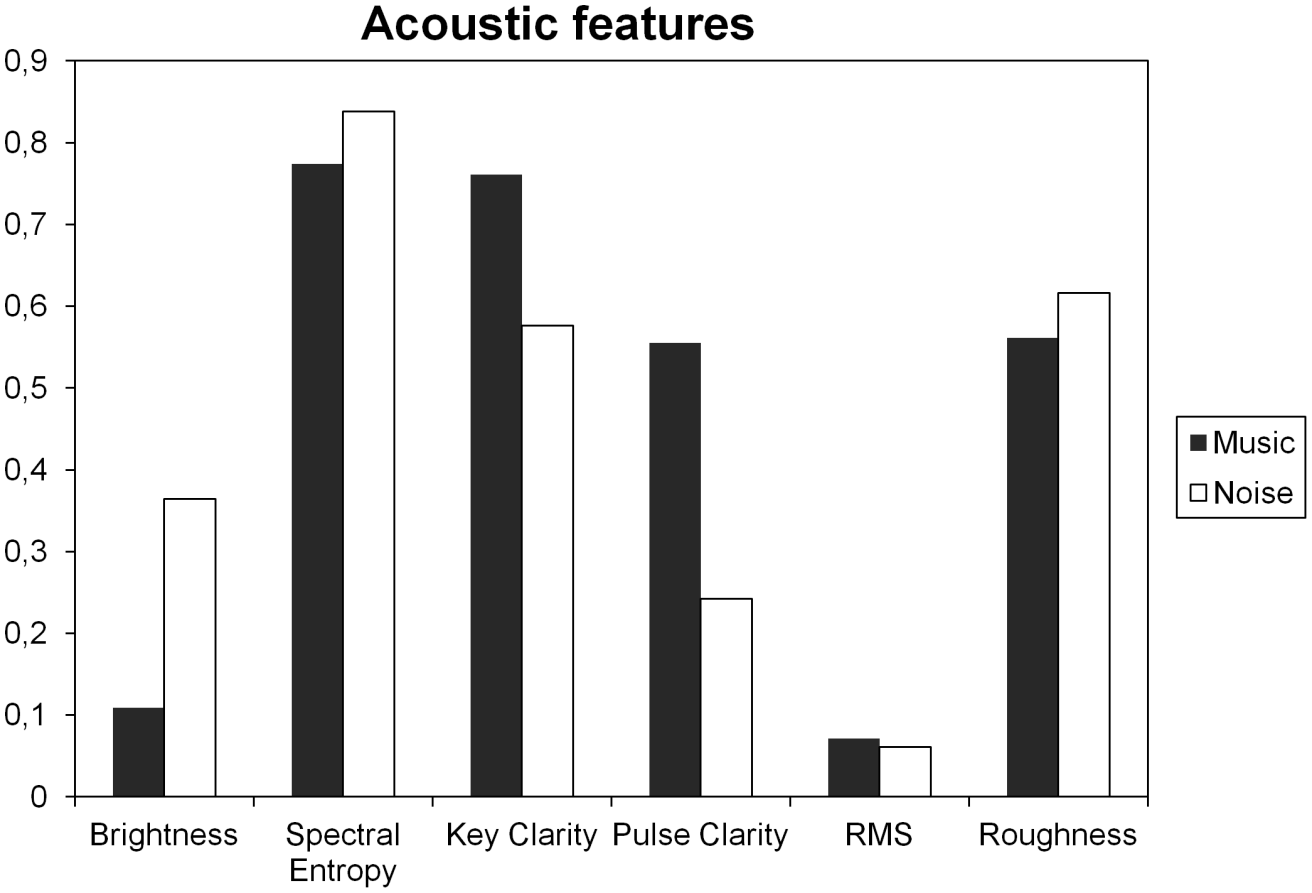
Acoustic features. Graph showing values of timbral (Brightness, RMS Energy and Roughness), rhythmic (Pulse Clarity) and tonal (Key Clarity) features of MusiCure and Noise extracted with MIRToolbox. MusiCure resulted to have comparable loudness than the Noise stimulus, but to be less bright, rougher and contain clearer tonal centers and pulse, as revealed by the mean parameters of Brightness, RMS, Key Clarity and Pulse Clarity, respectively.

The main timbral (Brightness, RMS Energy and Roughness), rhythmic (Pulse Clarity) and tonal (Key Clarity) features were extracted with the MIRToolbox from the sound sequences and used to determine the effects of these features on mood and emotional processing. The choice of features was made based on published results on the relationship between acoustic features (also extracted via MIRToolbox) and behavioral measures during music listening [45]–[51]. This analysis revealed that MusiCure and Noise stimulus had comparable loudness, but MusiCure turned out to be less bright, rougher and contain clearer tonal centers and pulse, as revealed by the mean parameters of Brightness, RMS, Key Clarity and Pulse Clarity, respectively (see Figure 1).

The sound stimuli were pre-tested informally within the lab personnel: four people out of five found MusiCure very relaxing and Noise very irritating. The participants of the study rated the two sound environments according to affective adjectives (happy, sad, arousing, pleasant, disgusting and irritating). The mood changes following the two sound sessions were assessed in our sample using the Italian version of the Profile of Mood State (POMS) questionnaire [52], [53]. Details about mood assessment and sound affective ratings are explained below in “Procedures”.

### Procedures

Prior to the experiment, all subjects completed the Big Five Questionnaire (BFQ) [54] and the State Trait Anxiety Index (STAI X2) [55]. The BFQ measures personality traits according to the Big Five Factors Model [56] and includes five dimensions (energy, friendliness, conscientiousness, emotional stability, and openness), which are organized into two facets each (energy: dynamism and dominance; friendliness: cooperativeness and politeness; conscientiousness: scrupulousness and perseverance; emotional stability: emotional control and impulse control; openness: openness to culture and openness to experience). The dimension “emotional stability” refers to aspects of “negative affectivity” [54]. Within this dimension, the facet “emotional control” is defined as the capacity to cope adequately with one's own anxiety and emotionality. The STAI X2 measures the Anxiety Index as a personality trait.

**Figure 2:**
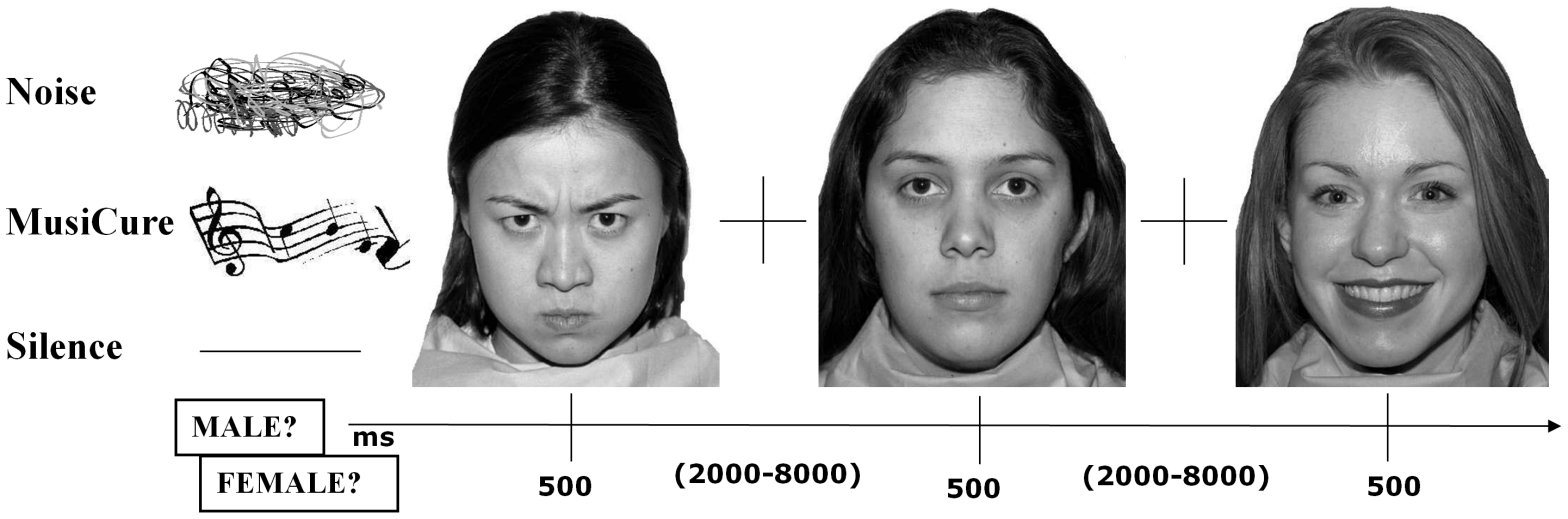
Neuropsychological task. Figure showing the implicit emotion-processing task. In this task, subjects were asked to identify the gender of angry, happy or neutral facial expressions while listening to a relaxing soundtrack (MusiCure) or while listening to amplitude-modulated noise or during silent background. Note: The images were taken from NimStim Face Stimulus Set (http://www.macbrain.org/resources.htm) with the permission of the authors.

The main task of the study was a computer-administered task on implicit emotion processing (Figure 2), in which blocks of mixed angry, happy, and neutral facial expressions from a validated set of facial pictures were presented (NimStim, http://www.macbrain.org/resources.htm) [57]. Subjects were asked to identify the gender of each face by a button press on the left or right arrows of the keyboard. The stimulus duration was 500 ms, and the interstimulus interval was randomly jittered between 2 and 8 s. 2 s were allowed for the button press response. The duration of each block was 5 min and 8 s. The total number of stimuli was 105: 30 angry, 37 happy, and 38 neutral faces. Each face was presented 1 to 3 times in each block. However, the same face containing the same facial expression was presented only once in each block. The order of the stimuli was randomized within and across the experimental conditions. A fixation crosshair was presented during the interstimulus interval.

Three different experimental conditions were defined by the sound background. In particular, subjects performed the implicit emotion processing task while listening to MusiCure (“MusiCure” condition), to the Noise sequence (“Noise” condition), or without sound background (“Silence” condition). The presentation of the three conditions was counterbalanced across subjects. Before starting each condition, 50 s of sound stimulation or silence were played with a fixation crosshair standing on the screen in order to induce potential mood effects. During the experiment, subjects wore headphones with closed-back earpieces for high ambient noise attenuation and signal isolation, with the volume set at 80 db.

In order to measure the mood changes, subjects answered to the Italian version of the POMS questionnaire [52], [53], which was presented on the screen before the task and after each condition. The POMS questionnaire included 58 items, pertaining to six mood state dimensions: Tension, Depression, Anger, Vigor, Fatigue, and Confusion.

Finally, during the MusiCure and Noise conditions, sound affective ratings were also acquired. In particular, subjects were asked to rate on a 5-point Likert scale if the sound background sounded happy, sad, arousing, pleasant, or disgusting. With regard to the POMS questionnaire and sound affective ratings, subjects answered by pressing the numbers from 1 to 5 on the top of the keyboard.

### Data analysis

Repeated-measures ANCOVAs were performed to investigate the association of sound conditions with mood state as measured by the POMS questionnaire at the beginning of the task and following each experimental block. For these ANCOVAs we used Sound Condition (before task, after MusiCure, after Noise, after Silence) as a repeated measures factor and the scores at each subscale of POMS as well as the Total Mood Disturbance (TMD) score as a dependent variable. According to the POMS scoring manual, the TMD score was calculated by summing the scores of the five negative mood scales (Tension, Depression, Anger, Fatigue and Confusion) and subtracting the Vigor scale. Higher TMD scores correspond to worse mood and more negative affective state. POMS data were not available for 5 subjects for technical reasons. For these individuals, POMS values were predicted by the expectation maximization (EM) algorithm [58]–[60].

**Figure 3:**
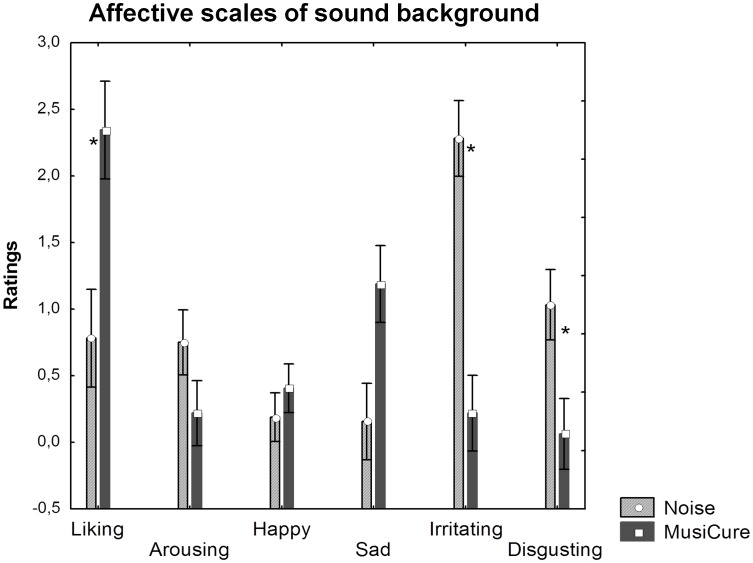
Affective scales of sound background. Graph showing ratings of affective scales of MusiCure and Noise background. Asterisks show statistical significance at p<0.05. Scales that had statistically significant difference between MusiCure and Noise also had significant positive correlation with RTs to the implicit emotional task (r = 0.6; p = 0.03).

An Affective Index of the sound backgrounds was used as a covariate of no interest in each analysis of variance conducted in this study. This procedure was made in order to avoid that the effect of sound backgrounds would just correspond to a measure of their perceived pleasantness, which is highly variable among individuals, and does not represent the focus of this study. The Affective Index was calculated by the sum of the sound affective ratings (liking, irritating and disgusting ratings) that were found to be significantly different between MusiCure and Noise (p<0.05) and that resulted to have a significant positive correlation with the behavioral responses during task conditions (r>0.6, p<0.03). Specifically, the sound affective ratings indicated that MusiCure was better liked than Noise, whereas Noise was rated as more irritating and disgusting than MusiCure (see Figure 3).

To study the association of sound-induced affective states and of individual affective traits with the speed of implicit emotion processing of facial expressions, we used a derived index of RT to the implicit emotion processing task as a dependent variable. In particular, since music processing is associated with more complex acoustic processing compared to noise, we calculated a RT index subtracting RT to neutral faces from RT to emotional faces (happy and angry faces). This procedure would allow us to control for the effect of different cognitive loads elicited by the two sound conditions. Specifically, to investigate the interaction between sound conditions and emotions, we carried out a 3×2 repeated measures analysis of covariance (ANCOVA) with Sound Condition (MusiCure, Noise, Silence) and Facial Emotion (Happy, Angry) as repeated measures factors.

To explore the interaction between trait anxiety, sound conditions and facial emotions on the derived RTs index during task performance, subjects were divided in two groups of high and low anxiety subjects based on the median z-score obtained with the STAI X2, consistent with other studies that have previously investigated this personality trait [61]. Thus, a multi-factorial ANCOVA was used, with Sound Condition (MusiCure, Noise and Silence), Facial Emotions (Happy, Angry) and Trait Anxiety as factors.

Similarly, the median z-score was used to divide subjects in those with low and high Emotional Control (EC) according to the BFQ. This procedure allowed us to investigate the interaction between emotional control scores at the BFQ, sound condition, and emotions on RTs during task performance. In addition, Fisher post hoc analyses were conducted to assess further differences amongst groups.

To investigate the contribution of the main acoustic features to the association between sound background effects and emotional as well as mood responses, we performed repeated measures ANCOVAs with Sound Condition as the independent factor (MusiCure, Noise) and new derived indexes of RTs and POMS scores as dependent variables. These indexes of RTs and POMS scores were obtained adding the mean timbral (Spectral Entropy, Brightness, RMS energy and Roughness), rhythmic (Pulse Clarity) and tonal (Key Clarity) features of MusiCure and Noise as constants to the behavioral measures of all individuals. We considered a decrease in the statistical effect of Sound Conditions on the adjusted RT and POMS scores as a measure of the contribution of a specific acoustic feature to that effect.

## Results

### Effects of sound condition on mood

**Figure 4:**
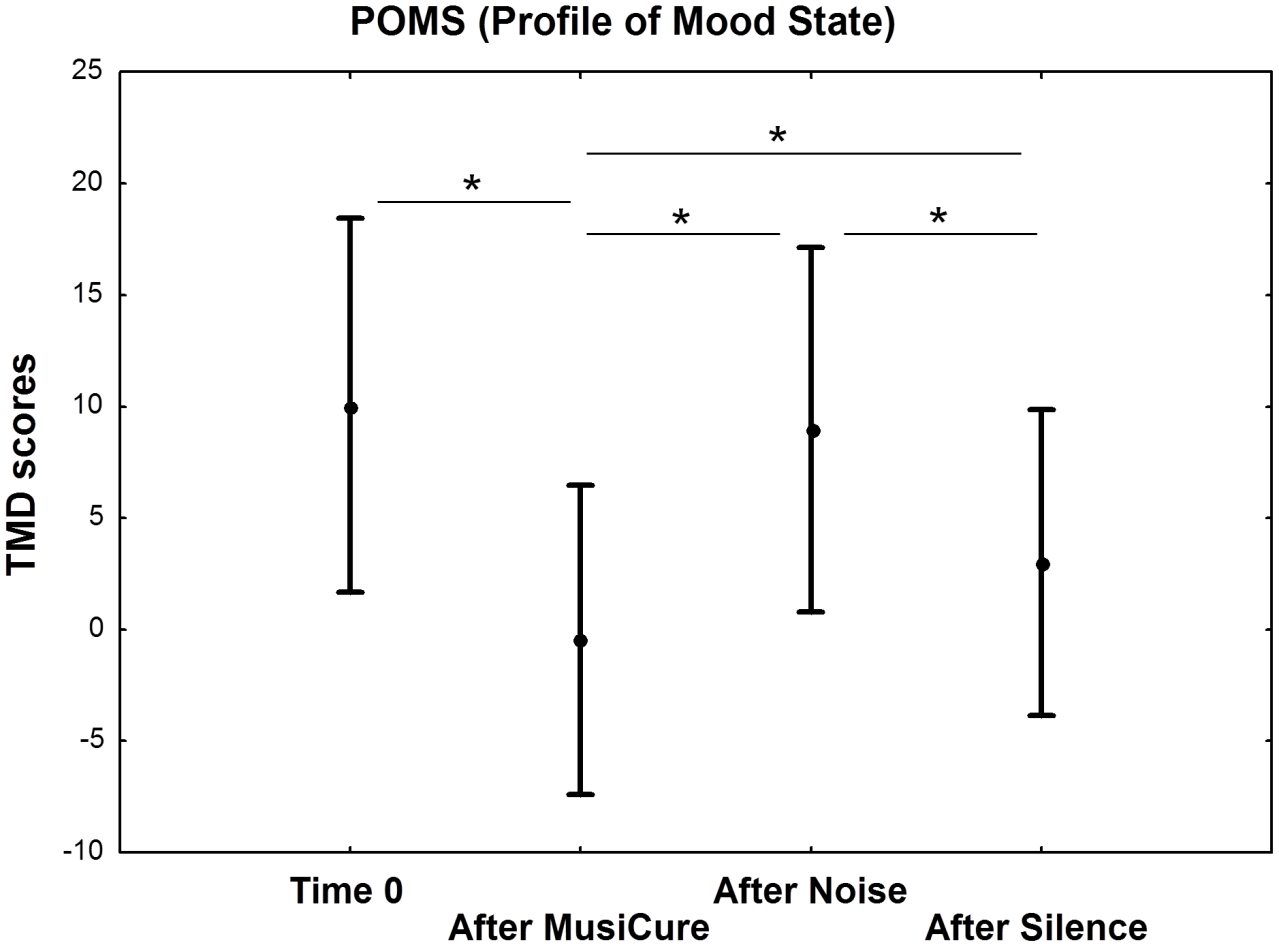
Profile of Mood State (POMS). Graph (mean ±0.95 confidence interval) showing a main effect of Sound Condition on TMD score of POMS questionnaire. Subjects revealed lower TMD scores after the MusiCure condition compared with all other experimental conditions, and greater TMD scores after the Noise condition compared with those after the Silence condition. Asterisks show statistical significance at p<0.05. See text for statistics.

Repeated-measures ANCOVA indicated a main effect of Sound Condition on POMS scores (F_3,93_ = 16.41, p<0.001). Fisher post-hoc tests revealed lower TMD scores after the MusiCure condition compared with baseline TMD scores measured at the beginning of the experimental session and with those obtained after the Noise and Silence conditions (all p<0.05). There was no statistically significant difference between TMD scores at baseline and after Noise (p = 0.9). However, the TMD scores were significantly greater after the Noise condition compared with those after the Silence condition (p = 0.007) (Figure 4). Analyses on POMS subscales revealed that the effect of MusiCure was more pronounced in the Fatigue scale, whereas the effect of Noise was more pronounced in the Confusion and Tension scales. There was no significant effect of Sound Condition in the Vigor scale (p>0.1).

### Effects of sound condition and emotion on behavior

**Figure 5:**
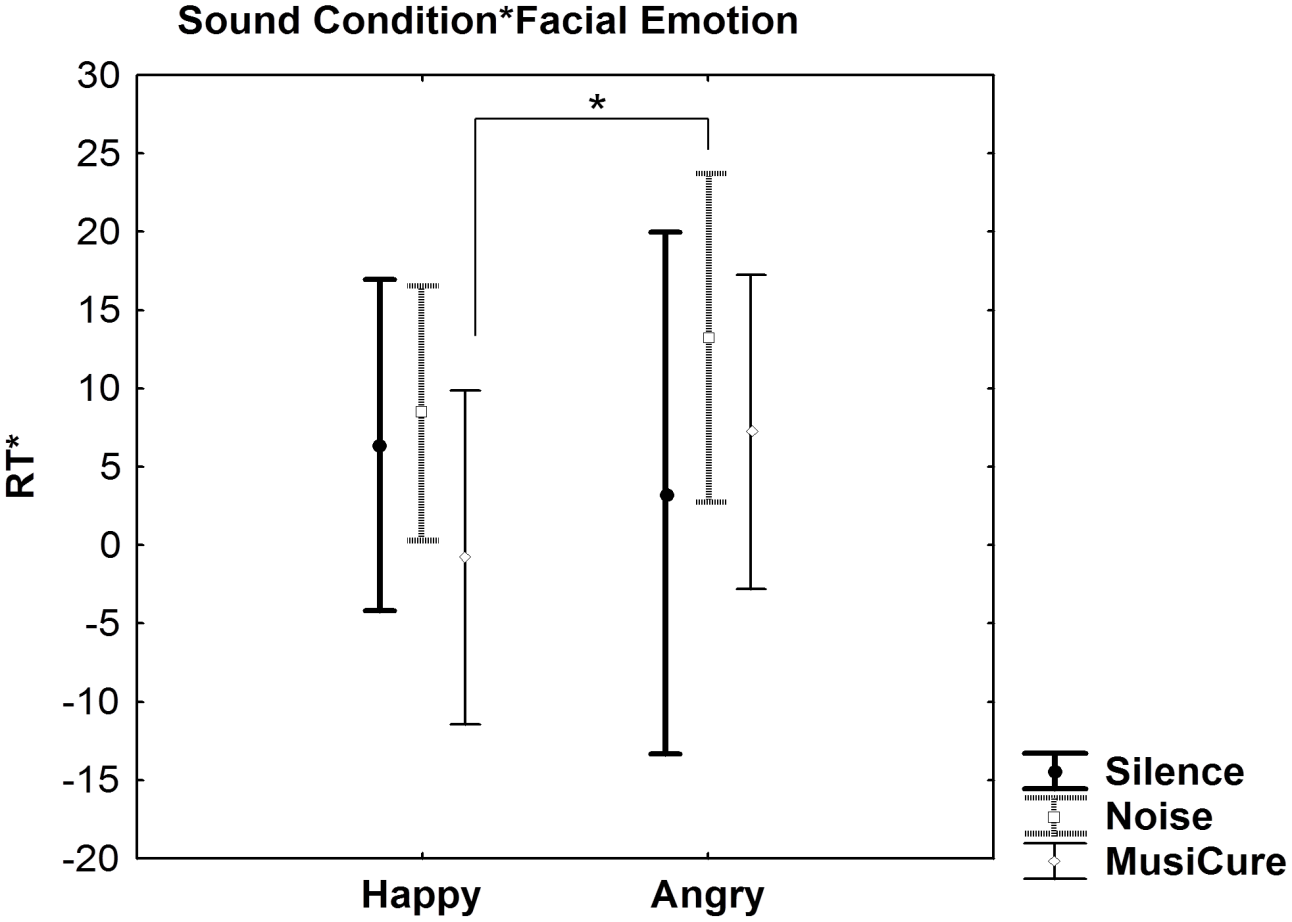
Sound condition by facial emotion. Graph (mean ±0.95 confidence interval) showing Sound Condition by Facial Emotion interaction on the derived index of RT during implicit processing of faces stimuli. Post hoc analyses revealed that this interaction results from faster responses to happy faces during the MusiCure condition compared with angry faces during the Noise condition. Asterisk shows statistical significance at p<0.05. See text for statistics.

Repeated-measures ANCOVA on reaction time data showed no main effect of Sound Condition or of Facial Emotion and a significant interaction between Sound Condition and Facial Emotion (F_2,60_ = 3.83, p<0.001). Post hoc analysis revealed that this interaction results from faster responses to happy faces during the MusiCure condition compared with angry faces during the Noise condition (p = 0.01). Post hoc analysis did not reveal any other statistically significant difference between sound and facial conditions (p>0.1) (Figure 5). No statistically significant effects were present on accuracy data (all p>0.1).

### The contribution of acoustic features on the sound effects on mood and behavior

Repeated measures ANCOVAs performed using the main acoustic features as constants, revealed that there was no effect of MusiCure and Noise if the Pulse Clarity values of each sound condition were added to the POMS scores of subjects (p = 0.8). Similarly, a 2 by 3 repeated measures ANCOVA revealed that there was no interaction between Sound Condition and Facial Emotion when the Pulse Clarity values of MusiCure and Noise were added to the RTs at the emotional task (p = 0.2). All the other acoustic features used as constants did not change the statistic significance of the sound effects on mood and behavior.

### Interaction between sound condition, emotion, and affective traits

The two groups that differed with respect to STAI X2 or EC scores were well matched in terms of age, gender, handedness and Hollingshead index (all p>0.2).

**Figure 6:**
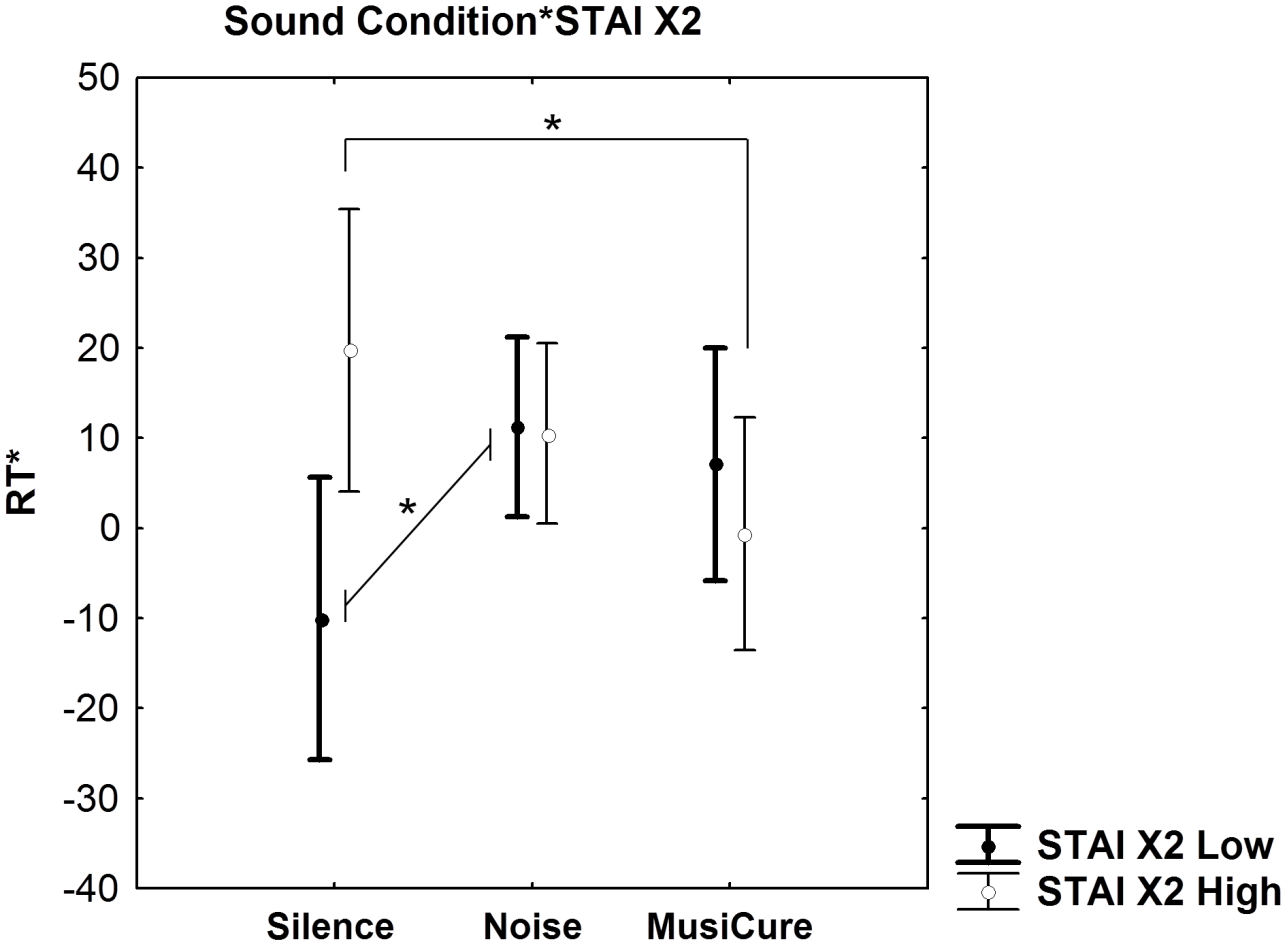
Sound condition by trait anxiety. Graph (mean ±0.95 confidence interval) showing Sound Condition by Trait Anxiety interaction on the derived index of the RT during implicit processing of faces stimuli. During Music, only individuals with greater Trait Anxiety had significant increases in their RTs when responding to faces in comparison to the Silence condition. During Noise only subjects with low trait anxiety had a significant reduction in RT compared with the Silence condition. Asterisks show statistical significance at p<0.05. See text for statistics.

A Multi-Factorial ANCOVA performed on RTs using STAI X2 as independent categorical factor, and Sound Condition as well as Facial Emotion as the within-group variables indicated a significant interaction between Trait Anxiety and Sound Condition (F_2,58_ = 4.62, p = 0.01). Post hoc analysis revealed that individuals with greater trait anxiety had faster RT during MusiCure, compared to the Silence condition (p = 0.02). On the other hand, low anxious subjects had slower RT during Noise compared to the Silence condition (p = 0.03). No other statistically significant difference was found at the post hoc analysis (p>0.1) (Figure 6). Moreover, no other main effects or interactions were present (all p>0.1). No statistically significant effects were present on accuracy data (all p>0.1).

**Figure 7:**
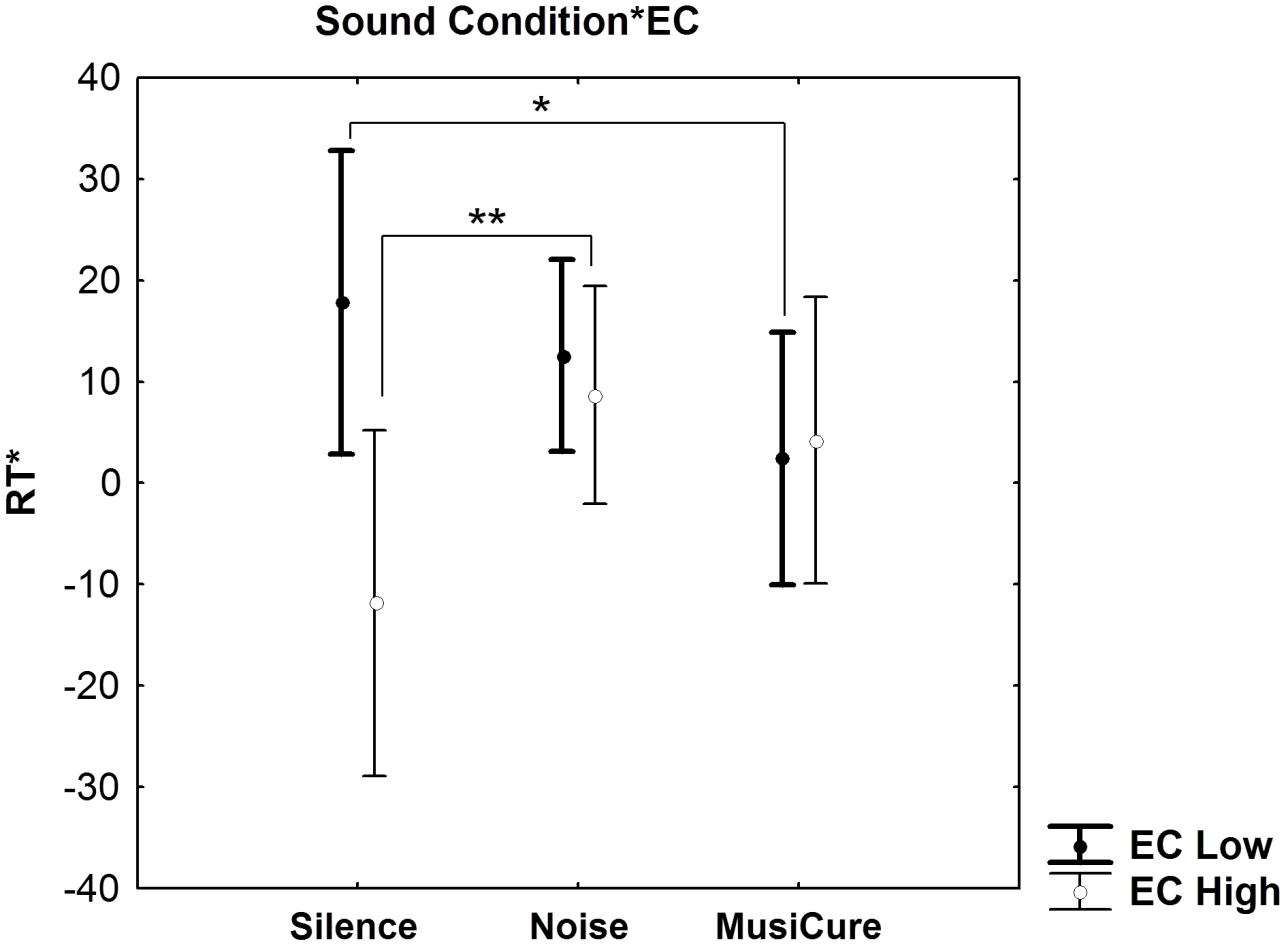
Sound condition by emotional control. Graph (mean ±0.95 confidence interval) showing Sound Condition by Emotional Control interaction on the derived index of RT during implicit processing of faces stimuli. During Music, only subjects with low EC had significant increases in RT when responding to faces in comparison to the Silence condition. During Noise, only individuals with high EC had greater RTs in comparison to the Silence condition (*p<0.05; **p<0.1). See text for statistics.

A Multi-Factorial ANCOVA performed on RTs using EC as categorical factor, and Sound Condition as well as Facial Emotion as the within-group variables indicated only a statistical trend for an interaction between EC and Sound Condition (F_2,58_ = 2.95, p = 0.056). Exploratory post-hoc analysis revealed that subjects with low EC had greater RT during MusiCure compared to the Silence condition (p = 0.05). Furthermore, individuals with high EC had greater RT during Noise compared to the Silence condition (p = 0.09). Post hoc analysis did not reveal any other statistical trend (p>0.2) (Figure 7). Moreover, no other main effects or interactions were present (all p>0.1). No statistically significant effects were present on accuracy data (all p>0.1).

## Discussion

In accordance with the well known effect of music in everyday affective regulation [22], we found that a 5-minutes excerpt of MusiCure, a relaxing musical piece including natural and environmental sounds, especially designed for therapeutic purposes, was successful in improving the affective state of healthy subjects in the laboratory, as indexed by the TMD score of the POMS questionnaire after MusiCure exposure compared with the TMD scores before and after each experimental session. Moreover, the adverse sound stimulus used in this study (Noise) changed the affective states negatively compared with the Silence and MusiCure condition. This result is in line with previous empirical findings of the negative impact of noise on the mood state, mostly provoking annoyance and anger [62]–[65]. Lack of a significant difference between TMD scores after Noise condition and before the task may rely on the non-neutral environment to which subjects were exposed just before entering the experimental room, which is located in a psychiatric hospital. However, in the Tension-Anxiety subscale of POMS questionnaire, the scores are significantly higher after Noise than before task and after MusiCure and Silence.

Our results indicate an interaction between the sound background and implicit processing of facial expressions. Subjects had faster reaction times during processing of happy faces in the MusiCure condition as compared with processing of angry faces during the irritating Noise condition. Previous studies [4]–[7] revealed that a temporary variation in affective state can modify the explicit, conscious labeling of emotional faces. Particularly, these studies suggest that individuals in an induced negative affective state label more stimuli as negative compared to positive or neutral stimuli, whereas those in a positive affective state tend to be more accurate in recognizing positive targets. Extending this evidence, results of our study indicated that changes in other kinds of affective states, induced by the sound environment, such as relaxed and tense ones, could affect emotional responses also during implicit processing of facial emotions. Thus, while a positive-oriented affective state elicited by MusiCure seems to facilitate the implicit emotional processing of positive, happy facial expressions, a negatively oriented affective state elicited by Noise acts in the opposite way during the implicit processing of angry faces. Considerable evidence from previous studies on implicit emotion processing suggests that humans tend to select negative stimuli more rapidly than positive stimuli [66]–[68]. In a visual searching task, Hansen and Hansen [69] found that participants picked out a lone angry face from a grid of happy faces more quickly than they picked out an happy face from a grid of angry faces, suggesting that the attention of subjects towards negative stimuli is rapidly and automatically captured. However, humans seem to have longer RTs in response to negative stimuli than to positive or neutral ones when they are asked to discriminate non-emotional features of the emotional stimuli [70], [71]. For example, in a behavioral study using an emotional Stroop color-naming task [71], color-naming latencies were longer for words with undesirable traits than for those with desirable traits. Thus, automatic vigilance may operate via preferential engagement [69] and delayed disengagement of attention [70]. That is, negative stimuli may attract more attention (preferential engagement) and hold attention longer (delayed disengagement) than neutral or positive stimuli [6]. The existence of this double mechanism for processing negative stimuli is related to their relevance in the surrounding world. However, the relevance of negative information may depend on several factors such as the mood state of individuals [72]. Thus, when threatening stimuli (angry faces) in this study were presented during the aversive Noise background, the negative emotional bias significantly affects RTs during gender discrimination of angry faces. In other words, here the negative mood induced by Noise (as evidenced by the POMS finding) modulated implicit processing of negative angry emotions. Such modulation possibly acts through re-directing and holding attentional resources to the threatening emotional information, which is more relevant in a negative mood context.

On the other hand, the relaxing soundtrack, MusiCure, shortened RTs to happy faces. Positive emotional faces *per se* do not need greater emotional load to be processed [73]. Hence, our results suggest that when happy faces occur in a positive mood context they engage even less emotional resources. However, our study does not directly investigate the emotional resources engaged by participants, but it indirectly derives them by their behavioral RTs. Thus, since the length of RTs can underlie several and distinct neural processes, alternative interpretations of our data cannot be excluded.

In this study we further investigated how individual affective dispositions, like trait anxiety and EC, are associated with behavioral responses at an implicit emotional task performed during experimental induction of affective states. Previous studies indicate that some affective states are more likely to be achieved by people with specific affective traits [12], [15]. In particular, Gray [14] suggested that extroverts and neurotics are differentially sensitive to stimuli that generate positive and negative affect, respectively. In support of this model, Larsen & Ketelaar [12] demonstrated that neurotic compared with non-neurotic subjects have heightened emotional reactivity to negative-mood induction, whereas extroverts compared with introverts show heightened emotional reactivity to positive-mood induction. In line with this literature, we found that individuals with greater trait anxiety (as assessed by the STAI X2) were faster in implicitly processing facial emotions during MusiCure than during Silence. In contrast, subjects with lower trait anxiety were slower in processing emotions during Noise than during Silence. These results suggest that high anxiety subjects are more sensitive to the emotion regulating effects of a relaxing soundtrack than those with lower anxiety rates. In contrast, subjects with lower anxiety rates are more affected by the Noise-induced negative effects on RT during the implicit processing of facial emotions, compared with high anxiety subjects. Similar results were present in the analysis of emotional control scores. Even if only at the trend level, subjects with a lower control of their emotions (as assessed by the EC subscale of the BFQ) had lower mean RTs while implicitly processing facial emotions during MusiCure than during Silence, whereas subjects with higher emotional control had higher mean RTs while implicitly processing emotions during Noise.

In the current study, two different sound stimulations have been used in order to induce opposite changes in the subjects' affective states and in their emotional responses to faces. These sound stimulations have been matched in amplitude and partially in pitch, their effects on affective states have been compared with two kinds of baseline conditions (before task and after Silence condition) and their effects on RTs to emotional faces have been filtered from bias through the use of an emotional baseline (neutral faces), besides the use of the Silence condition. Also, all the analyses have been covaried for the affective ratings of the sound stimuli in order to avoid that their effect would just correspond to a measure of their perceived pleasantness, which is highly variable among individuals, and does not represent the focus of this study. However, since MusiCure and Noise differ to each other in many acoustical aspects, we also extracted the main timbral, rhythmic and tonal acoustic features in order to understand which of them was more responsible of the sound effects found in this study. Results of these analyses revealed that for the effect of sound on affective states and emotional responses to faces, the rhythmic-related component (Pulse Clarity), which was higher in MusiCure than Noise, is considerably implicated. More specifically, if Pulse Clarity values are added to the analyses, the significant difference between MusiCure and Noise conditions disappears. Pulse clarity is considered as a high-level musical dimension that conveys how easily in a given musical piece, or a particular moment during that piece, listeners can perceive the underlying rhythmic or metrical pulsation [74]. One recent study, that used the same acoustic extraction method, found significant negative correlation between Pulse Clarity and emotion-related brain regions (e.s. amygdala and insula) [46]. According to the authors, low levels of perceived pulse may lead to higher tension and consequently higher activation of the limbic regions of the brain. However, in the mentioned study, the authors did not directly measure levels of tension in their subjects and this correlation may have a different interpretation. Nevertheless, our results, besides suggesting which of the acoustic component is responsible of the sound effects on mood and emotional responses to faces, may represent the behavioral effect of a rhythmic component previously related with changes in neural activity.

In conclusion, these data indicate that: 1. a 5-minutes sound environment can modify the affective state of subjects positively or negatively; 2. sound-induced positive and negative moods can alter the behavioral responses to angry and happy faces during an implicit processing task; 3. the sound effects on affective states and emotional responses to faces are mainly due to a rhythmic-related component of the sound stimuli. 4. individual anxiety partly explains the variability in processing emotions as well as the difference in the way relaxing or aversive sound environments impact this process. The results are evidence of beneficial effects of a relaxing soundtrack on the emotional life of more anxious subjects and their susceptibility to the adverse effects of a stressful sound environment.

## Supporting Information

Sound S1 **MusiCure excerpt.** The audio file includes 30-seconds excerpt of the MusiCure stimulus used in this study.
(WAV)

Sound S2 **Noise excerpt.** The audio file includes 30-seconds excerpt of the Noise stimulus used in this study.
(WAV)
